# Supplementary material for: Wnt activation disturbs cell competition and causes diffuse invasion of transformed cells through NF-κB-MMP21 pathway
Source: Nat Commun. 2023 Nov 3;14:7048. doi: 10.1038/s41467-023-42774-6 (PMC10624923; doi:10.1038/s41467-023-42774-6)
Supplement: Supplementary file 1 — Supplementary Information [file 41467_2023_42774_MOESM1_ESM.pdf]

Supplementary information for

Wnt activation disturbs cell competition and causes diffuse invasion of transformed cells through NF-κB-MMP21 pathway

Kazuki Nakai, Hancheng Lin, Shotaro Yamano, Shinya Tanaka, Sho Kitamoto, Hitoshi Saitoh, Kenta Sakuma, Junpei Kurauchi, Eilma Akter, Masamitsu Konno, Kojiro Ishibashi, Ryo Kamata, Akihiro Ohashi, Jun Koseki, Hirotaka Takahashi, Hideshi Yokoyama, Yukihiro Shiraki, Atsushi Enomoto, Sohei Abe, Yoku Hayakawa, Tetsuo Ushiku, Michihiro Mutoh, Yasuyuki Fujita & Shunsuke Kon.

Supplementary information
Supplementary Figures 1-10
Supplementary Tables 1-2

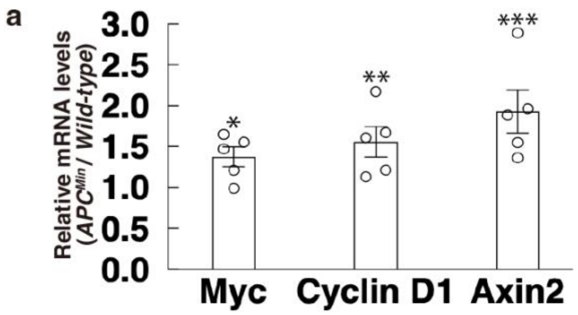

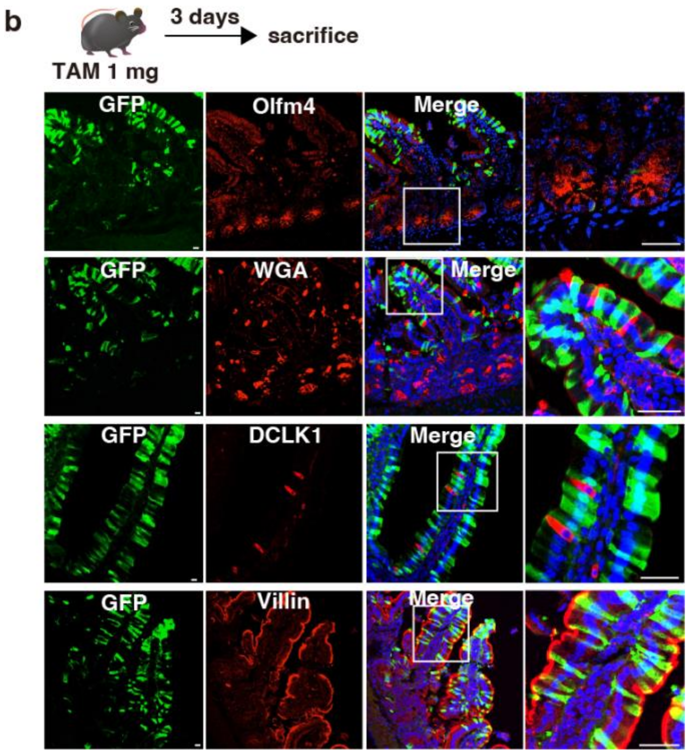

Supplementary Fig. 1. Wnt activation by *APC*-ablation and tamoxifen-induced expression of transgenes in a cell competition mouse model. (**a**) Effect of *APC*-ablation on mRNA levels of Wnt-target genes. mRNA was extracted from intestinal organoids genotyped as *wild-type* or APC^{Min} mice and subjected to q-PCR analyses. The primers used are listed in Supplementary Table 1. Values are shown as fold changes in APC^{Min} cells relative to *wild-type* cells. Data are mean ± s.e.m. *n* = five independent experiments. *P=0.0367; **P=0.0442; ***P=0.0248, unpaired two-tailed *t*-test. (**b**) Immunofluorescence images of intestinal villi from *Villin-GFP* mice 3 days after low-dose tamoxifen injection. The sliced sections were prepared, and were stained with DAPI (blue), GFP antibody (green) and Olfm4, WGA, DCLK1 or Villin antibody (red). Note that GFP expression is overlapped with Villin-expressing differentiated enterocytes, but not with other lineage markers. Scale bars, 50 µm.

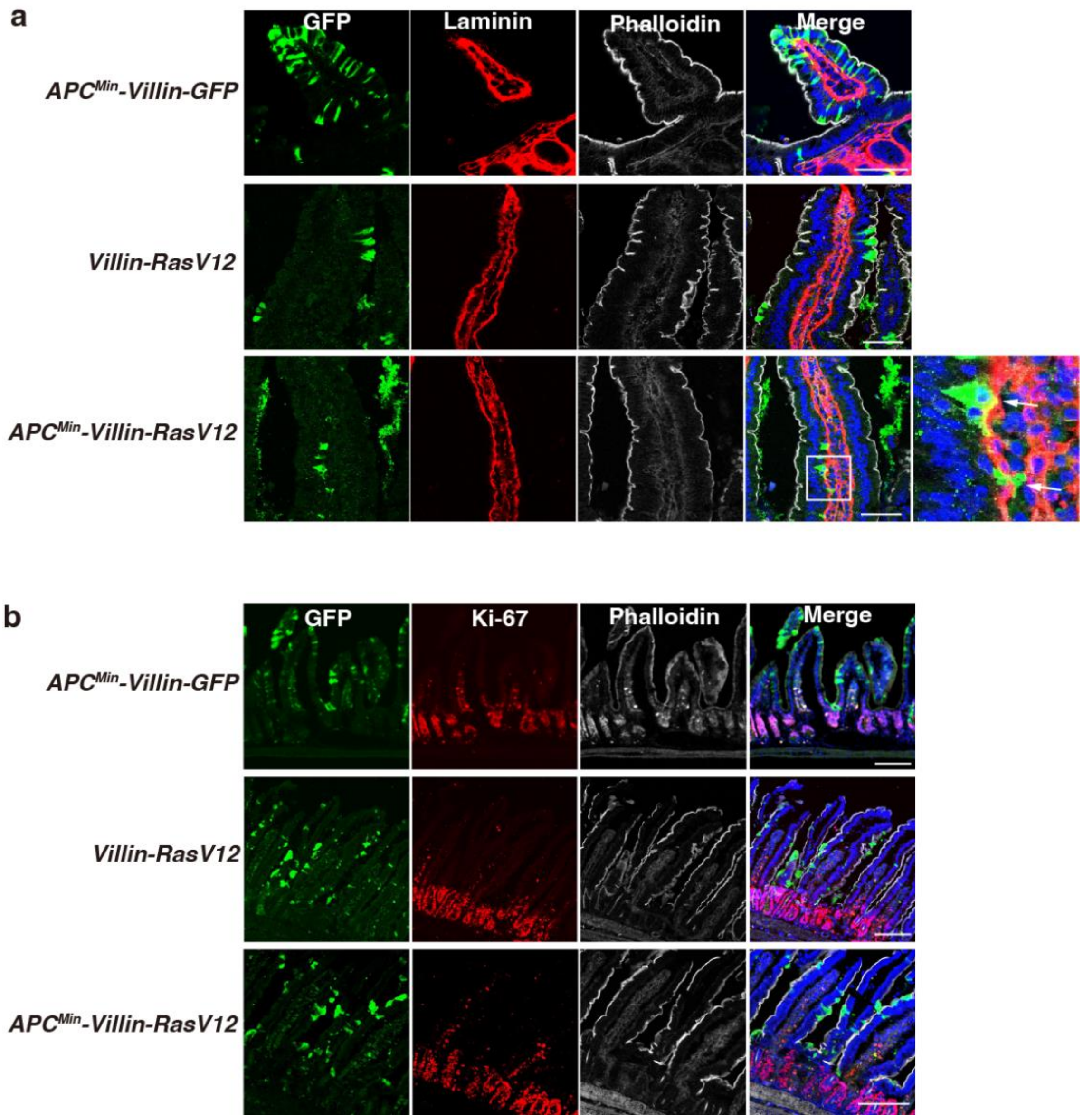

Supplementary Fig. 2. Invasive and proliferative capacity of APC^{Min}/RasV12-transformed cells. (**a**, **b**) Immunofluorescence images of laminin (**a**) or Ki-67 (**b**) in the small intestine of *APC^{Min}-Villin-GFP*, *Villin-RasV12* or *APC^{Min}-Villin-RasV12* mice at 3 days after 1 mg tamoxifen administration. The sliced sections were prepared, and were stained with DAPI (blue), GFP antibody (green), laminin antibody (**a**, red) or Ki-67 antibody (**b**, red) and phalloidin (white). Scale bars, 50 μm (**a**) and 100 μm (**b**). A white square in (**a**) highlights APC^{Min}/RasV12-transformed cells penetrating laminin-positive basement membrane (white arrows).

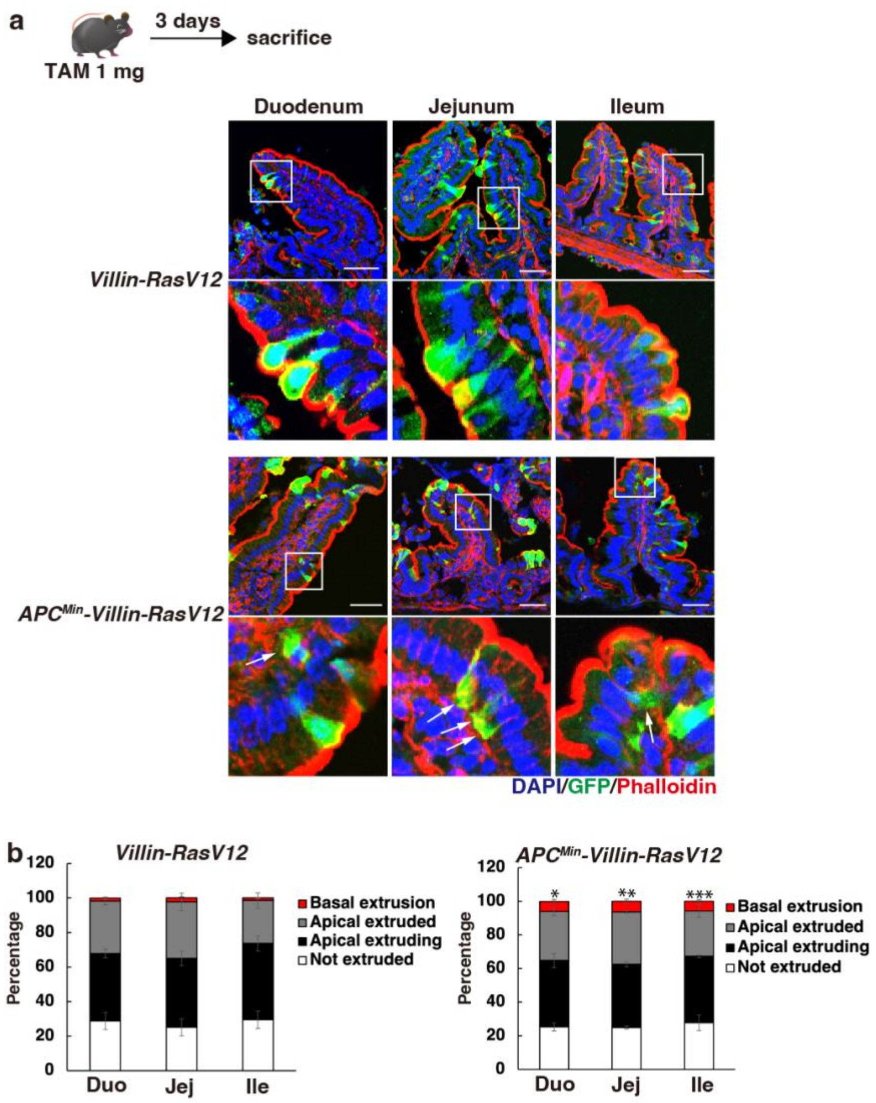

Supplementary Fig. 3. Basal extrusion of APC^{Min}/RasV12-transformed cells occurs at a similar frequency throughout the small intestine. (**a**) Confocal microscopic images of duodenum, jejunum or ileum from *Villin-RasV12* or *APC^{Min}-Villin-RasV12* mice. *Villin-RasV12* or *APC^{Min}-Villin-RasV12* mice were injected with 1 mg tamoxifen and were sacrificed 3 days later. Frozen sections were stained with DAPI (blue), anti-GFP antibody (green) and phalloidin (red). White squares in the top images are magnified in the corresponding panels below. White arrows depict the basally extruded cells. Scale bars, 50 μm. (**b**) Quantification of apical and basal extrusion of transformed cells in duodenum (Duo), jejunum (Jej) or ileum (Ile). Data are mean ± s.e.m. *n* = three independent experiments. **P*=0.0304; ***P*=0.0435; ****P*=0.0402 for basally extruded cells between RasV12- and APC^{Min}/RasV12-expressing cells, unpaired two-tailed *t*-test.

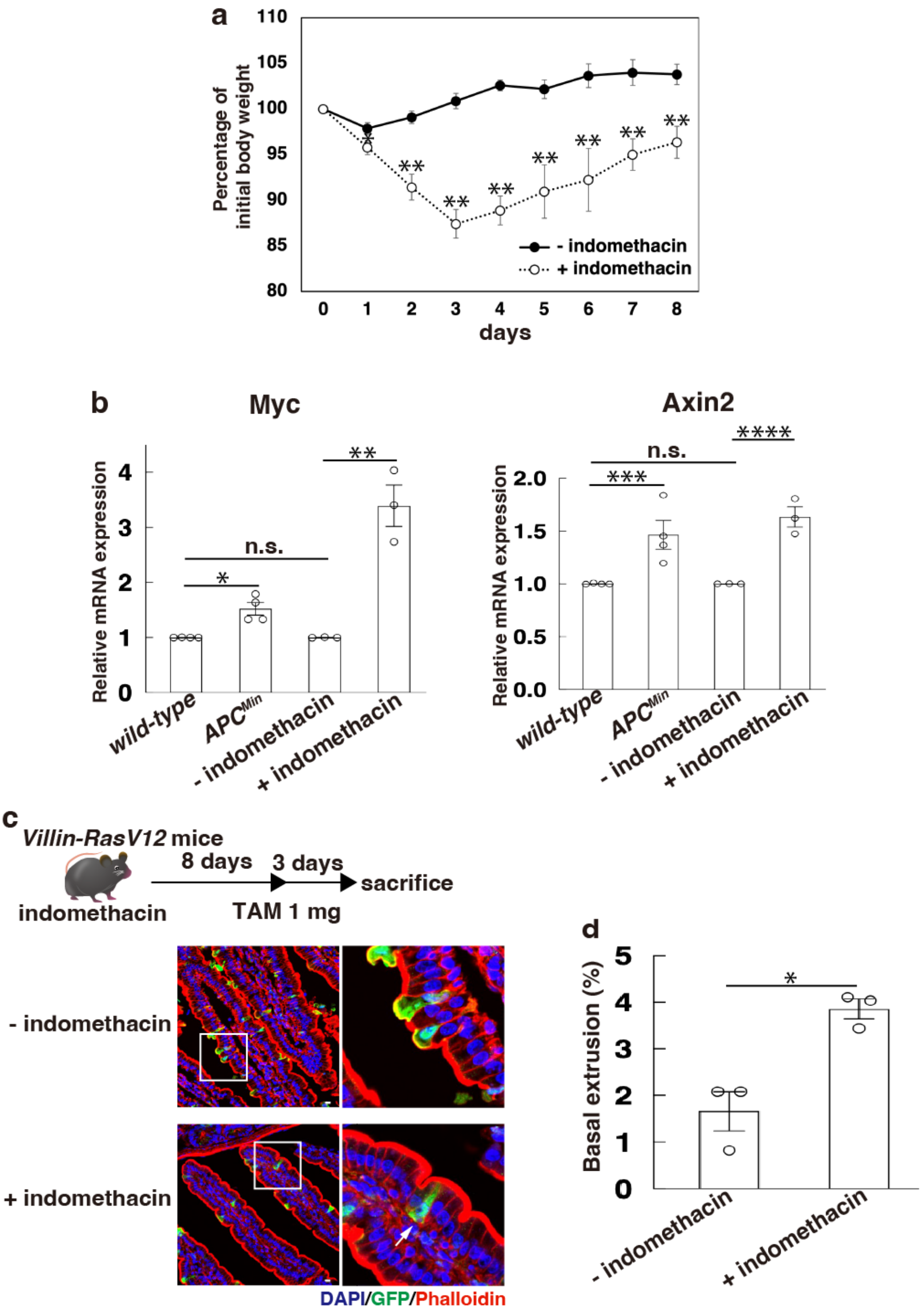
a
Percentage of initial body weight
days
- indomethacin
+ indomethacin
b
Myc
Axin2
Relative mRNA expression
n.s.
wild-type
APCMin
- indomethacin
+ indomethacin
c
Villin-RasV12 mice
indomethacin
8 days
3 days
TAM 1 mg
sacrifice
- indomethacin
+ indomethacin
DAPI/GFP/Phalloidin
d
Basal extrusion (%)
- indomethacin
+ indomethacin

Supplementary Fig. 4. Tissue injury-induced Wnt activation leads to basal extrusion of APC^{Min}/RasV12-transformed cells. (**a**) Effect of indomethacin administration on mouse body weight. Mice were subcutaneously administered either vehicle (5% $NaHCO_3$) or indomethacin (12.5 mg kg^{-1}) and weighed daily. Data are mean ± s.e.m. *n* = five independent experiments. **P*=0.0456; ***P*<0.01, unpaired two-tailed *t*-test. (**b**) Effect of tissue injury by indomethacin on mRNA levels of Wnt-target genes. mRNA was extracted from the small intestine of *wild-type*, *APC^{Min}*, or vehicle- or indomethacin-treated mice and subjected to q-PCR analyses. Fold changes relative to *wild-type* mice are presented. Data are mean ± s.e.m. *n* = three or four independent experiments. **P*=0.0199; ***P*=0.0238; ****P*=0.0424; *****P*=0.0223, unpaired two-tailed *t*-test. (**c**, **d**) Effect of indomethacin administration on the cell fates of RasV12-transformed cells. (**c**) Immunofluorescence images of intestinal villi from *Villin-RasV12* mice administered with vehicle or indomethacin. Magnified images depict apical or basal extrusion of transformed cells from vehicle- or indomethacin-administered mice, respectively. An arrow indicates a basally extruded cell. Scale bars, 10 μm (**c**). (**d**) Quantification of basal extrusion of RasV12-transformed cells in vehicle- or indomethacin-treated mice. Data are mean ± s.e.m. *n* = three independent experiments. **P*=0.0095 between the vehicle- and indomethacin-administrated group, unpaired two-tailed *t*-test.

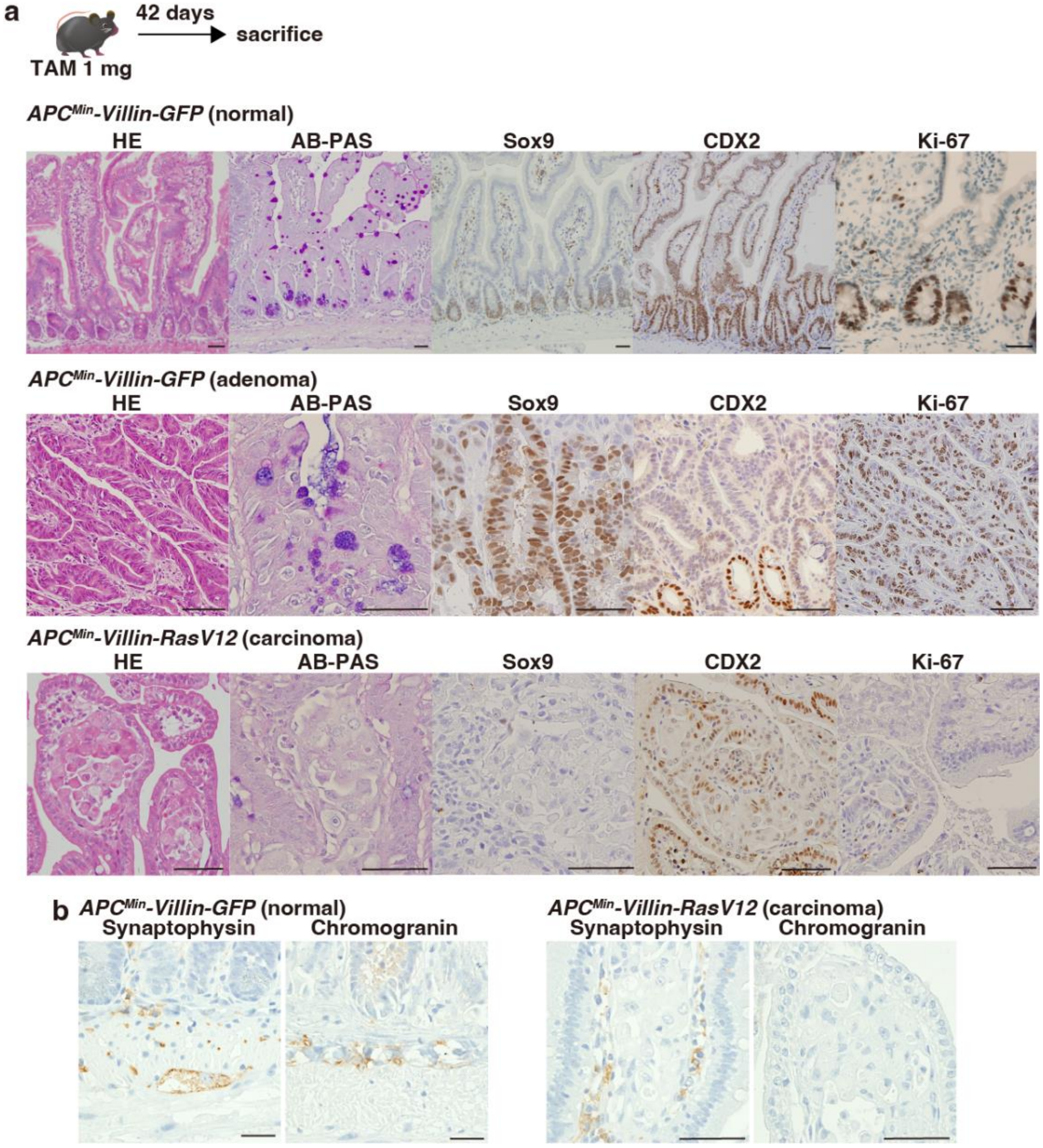

Supplementary Fig. 5. Characteristic profiling of APC^{Min}/RasV12-transformed carcinoma cells. (**a, b**) HE and immunostaining of intestinal villi from normal *APC^{Min}-Villin-GFP*, adenoma-bearing *APC^{Min}-Villin-GFP* or carcinoma-developing *APC^{Min}-Villin-RasV12* mice at 42 days after tamoxifen administration. Paraffin-embedded sections were processed for HE staining, or were stained with AB-PAS (purple), Sox9, CDX2 or Ki-67 antibody (brown) in (**a**). In (**b**), sections were stained with Synaptophysin or Chromogranin (brown) and were counterstained with methyl green. Scale bars, 50 μm (**a**, **b**).

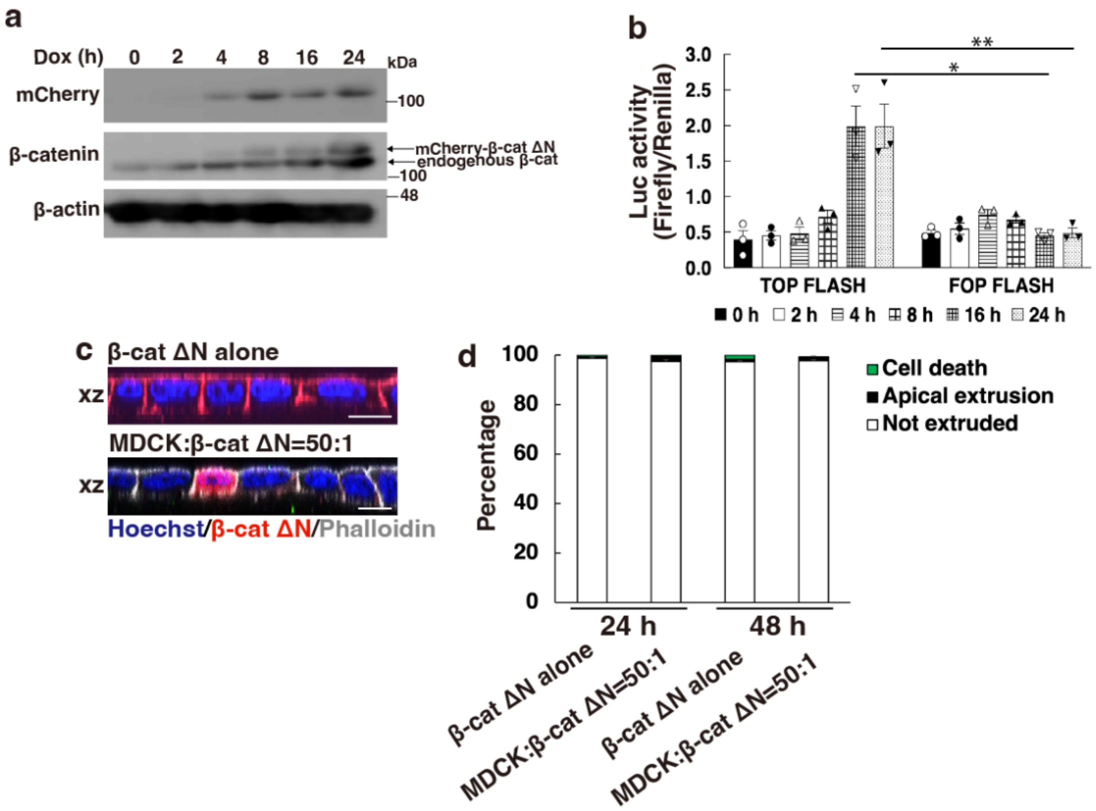

Supplementary Fig. 6. Wnt-activated cells are not eliminated by cell competition in MDCK cells. (**a**) Establishment of MDCK-pTRE3G mCherry-β-cateninΔ131 cells. Doxycycline-induced expression of mCherry-β-cateninΔ131 is determined by western blotting. (**b**) TOP FLASH reporter assay. MDCK-pTRE3G mCherry-β-cateninΔ131 cells were co-transfected with TOP FLASH or FOP FLASH and Renilla expressing vector, and were lysed and subjected to measurement of luciferase activity. Data are mean ± s.e.m. *n* = three independent experiments. **P*=0.0059; ***P*=0.0088, unpaired two-tailed *t*-test. (**c**) Immunofluorescence images of *xz* sections of MDCK-pTRE3G mCherry-β-cateninΔ131 cells cultured alone or surrounded by normal MDCK cells. Scale bars, 10 µm. (**d**) Quantification of the cell fates of MDCK-pTRE3G mCherry-β-cateninΔ131 cells cultured alone or surrounded by normal MDCK cells at 24 or 48 h after doxycycline addition. Data are mean ± s.e.m. *n* = three independent experiments.

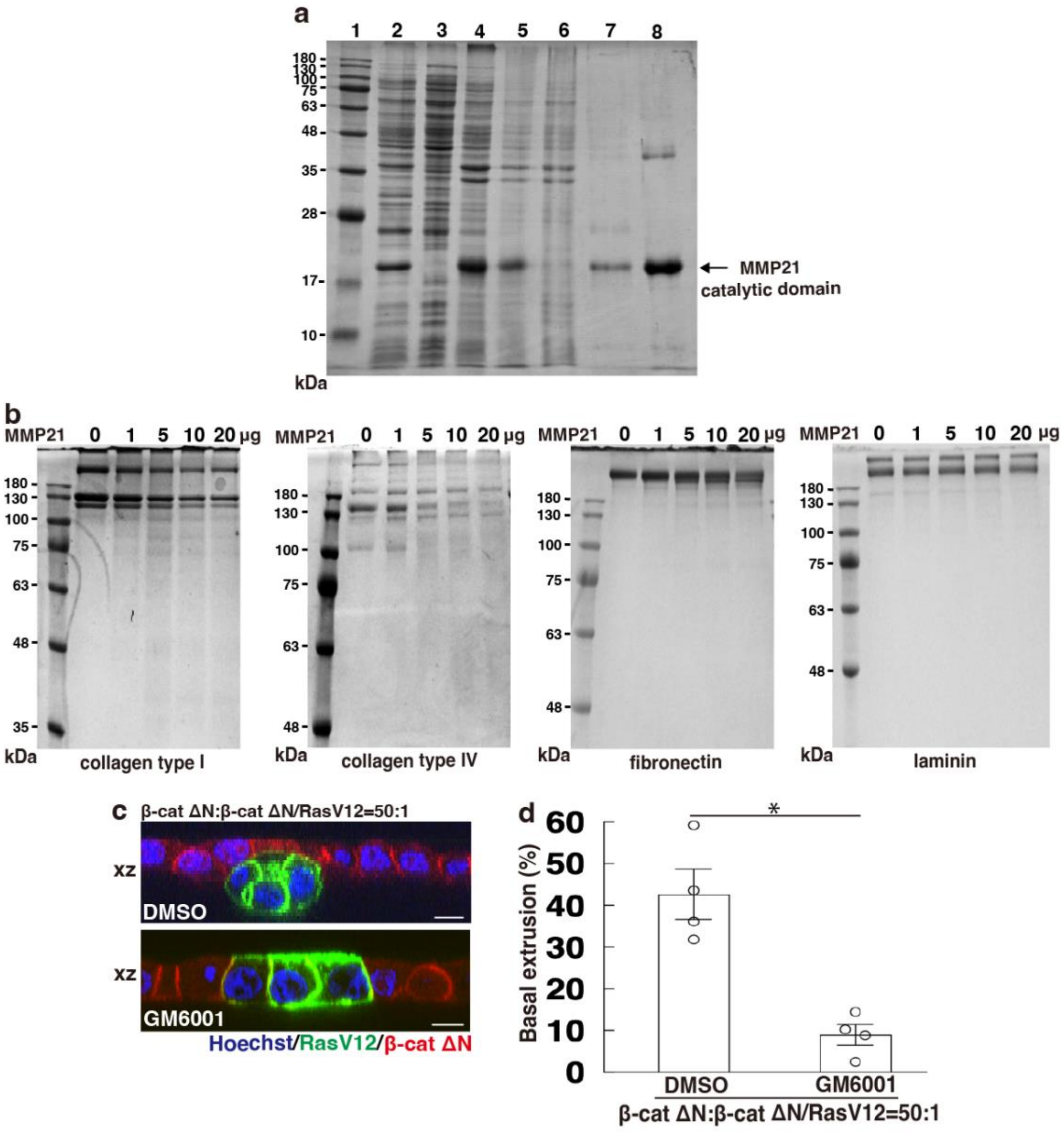
a
1 2 3 4 5 6 7 8
180 130 100 75 63 48 35 28 17 10
kDa
MMP21
catalytic domain
b
MMP21 0 1 5 10 20 μg
collagen type I
collagen type IV
fibronectin
laminin
kDa
c
β-cat ΔN:β-cat ΔN/RasV12=50:1
xz
DMSO
GM6001
Hoechst/RasV12/β-cat ΔN
d
Basal extrusion (%)
0 10 20 30 40 50 60
*
DMSO
GM6001
β-cat ΔN:β-cat ΔN/RasV12=50:1

Supplementary Fig. 7. MMP21 cleaves several extracellular matrix substrates and regulates basal extrusion of transformed cells. (**a**) Purification of MMP21 catalytic domain. A SDS-PAGE image shows purification process from the lysate to the final product. Lanes: 1, molecular marker; 2, induced protein extract; 3, soluble fraction before guanidine hydrochloride treatment; 4, insoluble fraction before guanidine hydrochloride treatment; 5, soluble fraction after guanidine hydrochloride treatment; 6, flow-through fraction from Ni-NTA resin; 7, wash-out fraction from Ni-NTA resin; 8, elute from Ni-NTA resin by 500 mM imidazole. The molecular mass of MMP21 catalytic domain-His is indicated by an arrow. (**b**) Degradation of ECM substrates by the MMP21 catalytic domain. Collagen type I, collagen type IV, fibronectin and laminin were incubated with buffer alone or with 1, 5, 10 or 20 μg of the MMP21 catalytic domain. The digestion products were analyzed by SDS-PAGE under reducing condition and stained with Coomassie Blue after electrophoresis. The sizes of molecular weight markers are shown to the left. (**c**) Confocal images of *xz* sections of MDCK-pTR GFP-RasV12 mCherry-β-cateninΔ131 cells surrounded by MDCK mCherry-β-cateninΔ131 cells in the absence or presence of 25 μM GM6001. Scale bars, 10 μm. (**d**) Quantification of basal extrusion of β-cat ΔN/RasV12 cells in the absence or presence of GM6001. Data are mean ± s.e.m. n = four independent experiments. *P=0.0021, unpaired two-tailed t-test.

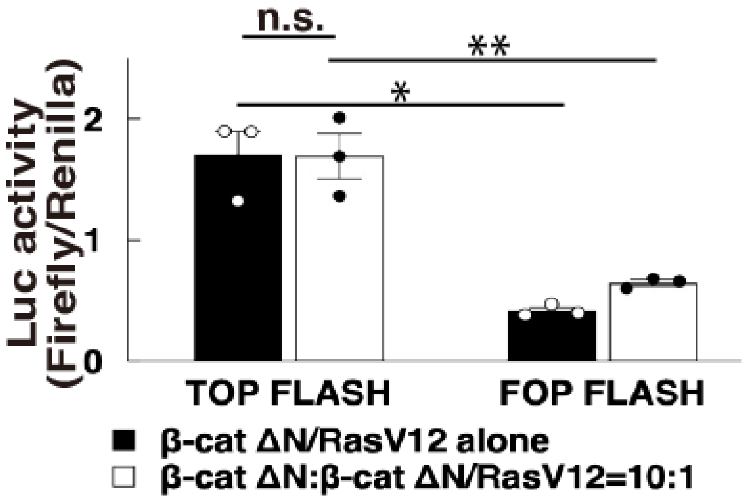

Supplementary Fig. 8. Wnt activity is not changed in β-cat ΔN/RasV12 cells surrounded by β-cat ΔN cells. MDCK-pTR GFP-RasV12 mCherry-β-cateninΔ131 cells were co-transfected with TOP FLASH or FOP FLASH and Renilla expressing vector, and were cultured alone or co-cultured with MDCK mCherry-β-cateninΔ131. After lysis, cells were subjected to measurement of luciferase activity. Data are mean ± s.e.m. *n* = three independent experiments. **P*=0.0197; ***P*=0.0291, unpaired two-tailed *t*-test.

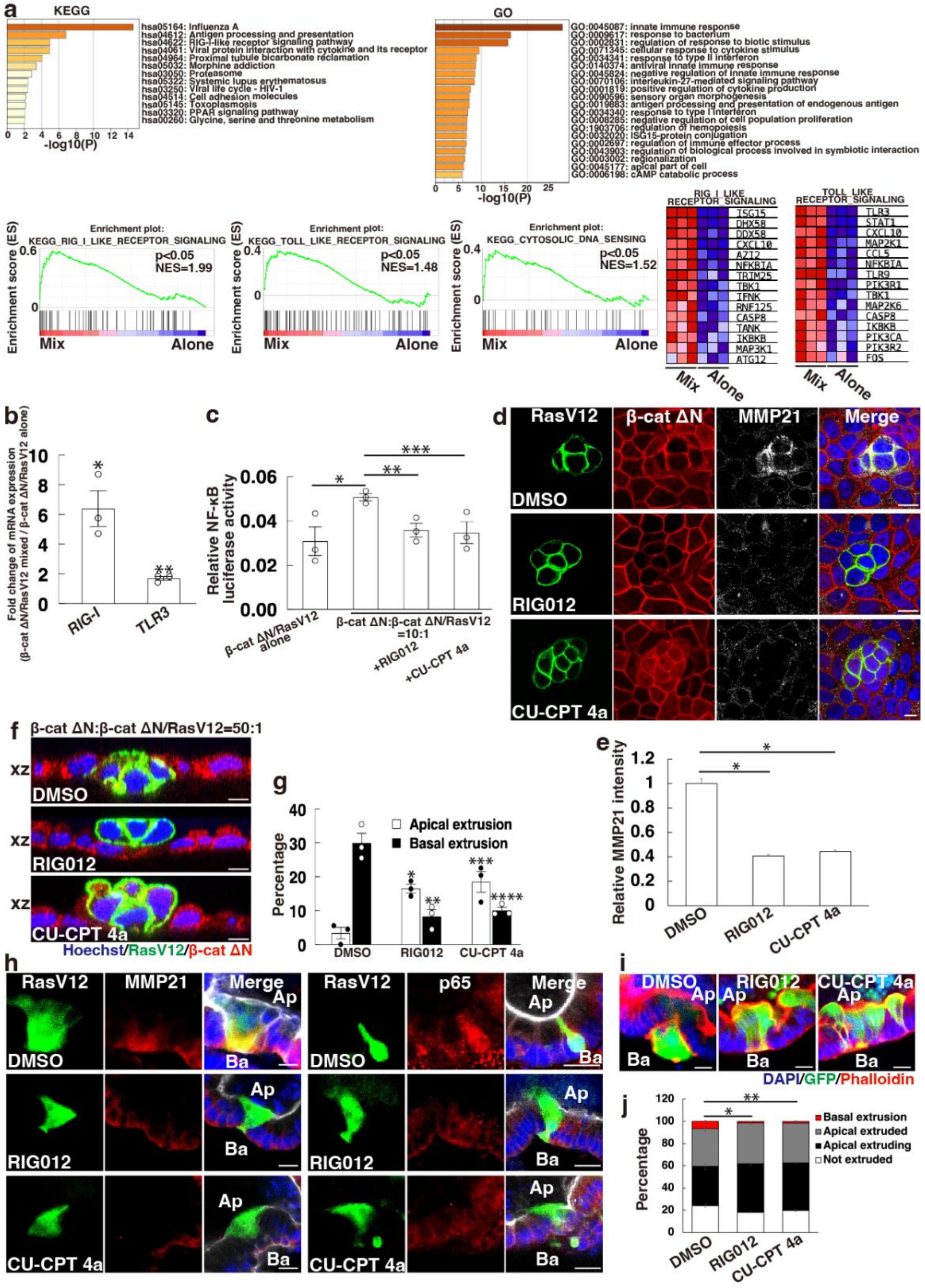
a
KEGG
hsa05164: Influenza A
hsa04612: Antigen processing and presentation
hsa04622: RIG-I-like receptor signaling pathway
hsa04061: Viral protein interaction with cytokine and its receptor
hsa04964: Proximal tubule bicarbonate reclamation
hsa05032: Morphine addiction
hsa03050: Proteasome
hsa05322: Systemic lupus erythematosus
hsa03250: Viral life cycle - HIV-1
hsa04514: Cell adhesion molecules
hsa05145: Toxoplasmosis
hsa03320: PPAR signaling pathway
hsa00260: Glycine, serine and threonine metabolism
-log10(P)
GO
GO:0045087: innate immune response
GO:0009617: response to bacterium
GO:0002831: regulation of response to biotic stimulus
GO:0071345: cellular response to cytokine stimulus
GO:0034341: response to type II interferon
GO:0140374: antiviral innate immune response
GO:0045824: negative regulation of innate immune response
GO:0070106: interleukin-27-mediated signaling pathway
GO:0001819: positive regulation of cytokine production
GO:0090596: sensory organ morphogenesis
GO:0019883: antigen processing and presentation of endogenous antigen
GO:0034340: response to type I interferon
GO:0008285: negative regulation of cell population proliferation
GO:1903706: regulation of hemopoiesis
GO:0032020: ISG15-protein conjugation
GO:0002697: regulation of immune effector process
GO:0043903: regulation of biological process involved in symbiotic interaction
GO:0003002: regionalization
GO:0045177: apical part of cell
GO:0006198: cAMP catabolic process
-log10(P)
Enrichment plot: KEGG_RIG_I_LIKE_RECEPTOR_SIGNALING
p<0.05
NES=1.99
Enrichment plot: KEGG_TOLL_LIKE_RECEPTOR_SIGNALING
p<0.05
NES=1.48
Enrichment plot: KEGG_CYTOSOLIC_DNA_SENSING
p<0.05
NES=1.52
Enrichment score (ES)
Mix
Alone
RIG_I_LIKE RECEPTOR_SIGNALING
ISG15
DHX58
DDX58
CXCL10
AZI2
NFKBIA
TRIM25
TBK1
IFNK
RNF125
CASP8
TANK
IKBKB
MAP3K1
ATG12
TOLL_LIKE RECEPTOR_SIGNALING
TLR3
STAT1
CXCL10
MAP2K1
CCL5
NFKBIA
TLR9
PIK3R1
TBK1
MAP2K6
CASP8
IKBKB
PIK3CA
PIK3R2
FOS
b
Fold change of mRNA expression (β-cat ΔN/RasV12 mixed / β-cat ΔN/RasV12 alone)
RIG-I
TLR3
c
Relative NF-κB luciferase activity
β-cat ΔN/RasV12 alone
β-cat ΔN:β-cat ΔN/RasV12 =10:1
+RIG012
+CU-CPT 4a
d
RasV12
β-cat ΔN
MMP21
Merge
DMSO
RIG012
CU-CPT 4a
e
Relative MMP21 intensity
DMSO
RIG012
CU-CPT 4a
f
β-cat ΔN:β-cat ΔN/RasV12=50:1
xz
DMSO
RIG012
CU-CPT 4a
Hoechst/RasV12/β-cat ΔN
g
Percentage
Apical extrusion
Basal extrusion
DMSO
RIG012
CU-CPT 4a
h
RasV12
MMP21
Merge
p65
Ap
Ba
DMSO
RIG012
CU-CPT 4a
i
DMSO
RIG012
CU-CPT 4a
Ap
Ba
DAPI/GFP/Phalloidin
j
Percentage
Basal extrusion
Apical extruded
Apical extruding
Not extruded
DMSO
RIG012
CU-CPT 4a

Supplementary Fig. 9. Innate immune response governs the basal extrusion of transformed cells via NF-κB-MMP21 pathway. (**a**) KEGG and GO enrichment analyses (upper), showing the top clusters colored according to the *P*-value. GSEA plots (lower left) showing a significant correlation between the transcriptional profile of β-cat ΔN/RasV12 cells co-cultured with β-cat ΔN cells and RIG-I, TLR or DNA sensing pathway gene signatures. Heatmap shows top 15 marker genes for RIG-I or TLR signaling (lower right). Expression values are represented as colors and range from red to blue based on the expression level. (**b**) q-PCR analysis of RIG-I and TLR3 in β-cat ΔN/RasV12 cells surrounded by β-cat ΔN cells. β-cat ΔN/RasV12 cells were co-cultured with β-cat ΔN cells or cultured alone, and subjected to q-PCR analysis. Values are shown as fold change in β-cat ΔN/RasV12 cells surrounded by β-cat ΔN cells relative to β-cat ΔN/RasV12 cells cultured alone. Data are mean ± s.e.m. *n* = three independent experiments. **P*=0.0460; ***P*=0.0395, unpaired two-tailed *t*-test. (**c**-**g**) Effect of RIG012 or CU-CPT 4a on NF-κB activity, MMP21 expression and cell fates of β-cat ΔN/RasV12 cells. (**c**) NF-κB reporter assay. β-cat ΔN/RasV12 cells were co-transfected with NF-κB luciferase reporter and Renilla expressing vector, and were co-cultured with β-cat ΔN cells or cultured alone in the absence or presence of 2.5 μM RIG012 or 50 μM CU-CPT 4a. After lysis, cells were subjected to measurement of luciferase activity. Data are mean ± s.e.m. *n* = three independent experiments. **P*=0.0407; ***P*=0.0364; ****P*=0.0133, unpaired two-tailed *t*-test. (**d**) Immunofluorescence images of MMP21 in the absence or presence of RIG012 or CU-CPT 4a. β-cat ΔN/RasV12 cells were mixed with β-cat ΔN cells in the presence of BFA, and were treated with DMSO, RIG012 or CU-CPT 4a. Cells were stained with Hoechst 33342 (blue) and anti-MMP21 antibody (white). (**e**) Quantification of fluorescence intensity of MMP21. Values are expressed as a ratio relative to DMSO treatment. Data are mean ± s.e.m. **P*<0.001, unpaired two-tailed *t*-test; *n* = 364, 396 and 445 cells pooled from three independent experiments. (**f**) Immunofluorescence images of *xz* sections of MDCK-pTR GFP-RasV12 mCherry-β-cateninΔ131 cells surrounded by MDCK mCherry-β-cateninΔ131 cells in the absence or presence of RIG012 or CU-CPT 4a. (**g**) Quantification of apical and basal extrusion in the absence or presence of RIG012 or CU-CPT 4a. Data are mean ± s.e.m. *n* = three independent experiments. **P*=0.0046; ***P*=0.0055; ****P*=0.0208; *****P*=0.0144, unpaired two-tailed *t*-test. (**h**-**j**) Effect of innate immunity inhibition on MMP21, p65 expression and the cell fates *ex vivo*. (**h**) Immunofluorescence images of MMP21 or p65 in intestinal organoids from APC^{Min}*-Villin-RasV12* mice in the absence or presence of 10 μM RIG012 or 100 μM CU-CPT 4a. (**i**) Immunofluorescence images of intestinal organoids treated with 100 nM tamoxifen and cultured for 24 h in the absence or presence of RIG012 or CU-CPT 4a. Ba and Ap stand for the basal and apical sides, respectively (**h**, **i**). Scale bars, 10 μm (**d**, **f**, **h**, **i**). (**j**) Quantification of apical and basal extrusion. Data are mean ± s.e.m. *n* = three independent experiments. **P*=0.0233; ***P*=0.0150 for basally extruded cells between DMSO- and RIG012- or CU-CPT 4a-treated group, unpaired two-tailed *t*-test.

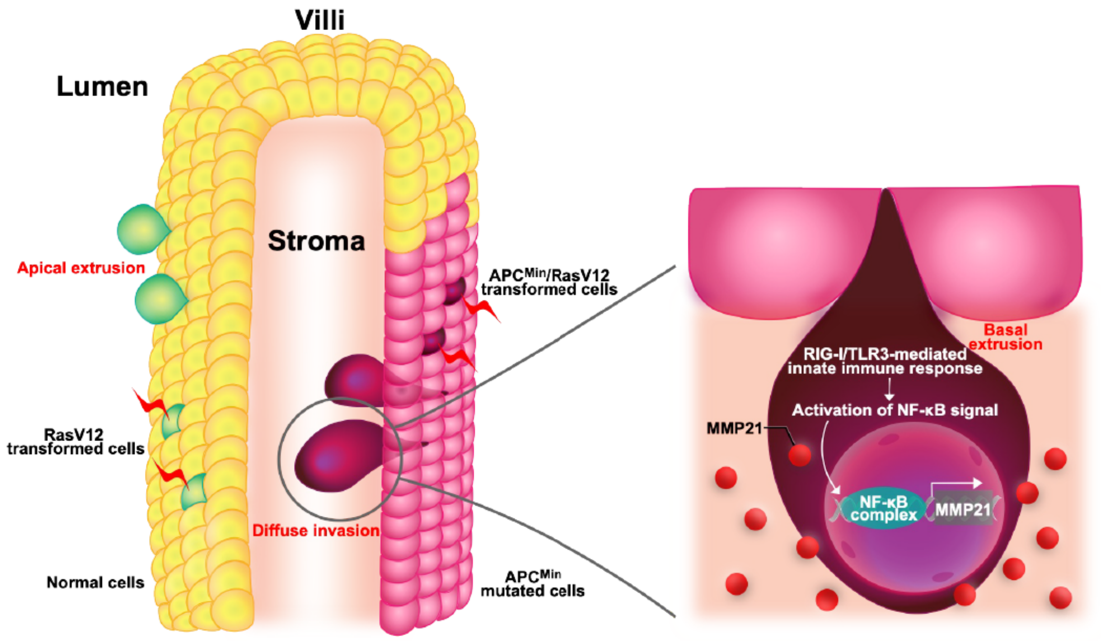

Supplementary Fig. 10. A schematic model for molecular mechanisms of the diffuse invasion of APC^{Min}/RasV12-transformed cells.

Supplementary Table 1. Primers used for quantitative real-time PCR analysis for Wnt activity of mice

Gene	Forward primer	Reverse primer
ACTINb	CATTGCTGACAGGATGCAGAAGG	TGCTGGAAGGTGGACAGTGAGG
Myc	GCCCAGTGAGGATATCTGGA	ATCGCAGATGAAGCTCTGGT
Cyclin D1	GGCACCTGGATTGTTCTGTT	CAGCTTGCTAGGGAACTTGG
Axin2	GGGGGAAAACACAGCTTACA	TTGACTGGGTCGCTTCTCTT

Supplementary Table 2. Primers used for q-PCR analysis for β-cat ΔN/RasV12 cells

Gene	Forward primer	Reverse primer
ACTINb	GGCACCCAGCACAATGAAG	ACAGTGAGGCCAGGATGGAG
MMP1	TTCGGGGAGAAGTGATGTTC	CTGACCCTGAACAACCCAGT
MMP2	CCACTATTGGTGGGAACTCG	TGCGACCAGGAATAAGCTGT
MMP3	CCAAGTGGAGGAAAACTCACC	CCAGGTCCATCAAAAGGGTTA
MMP7	ATCCATTTGATGGACCAGGA	GTCGGATACATCACGGCATC
MMP9	ATCTTCCTGGGCAAGCAGTA	TGGCACCGATGAATGATCTA
MMP11	CTAACGAGATTGCCCCACTG	CCCAATACTGAGCACCCTGA
MMP13	AGGTGCTTGTCTGGGAGAGA	GCTCTGCAAGCTGGAAGTCT
MMP14	TGCCCAATGGAAAGACCTAC	CATCACTGCCCATGAATGAC
MMP15	GACATCAGTGCTGCCTACGA	CGCTGTGTCTCCTCATTGAA
MMP17	AGCCTTGCAGAGGACACACT	TTGAGTTCTGCGTGATCCAG
MMP19	GCCATAGGAACAACCCTGAA	GTGGCTAGTGCTGGGACATT
MMP21	ACGGGGATCCTATCCAAATC	GGCCTGATCCTTGTCACTGT
MMP23B	GGCCAGAAGATCCTTCACAA	ACTCGCCAGGAGTAGGTGCT
MMP26	ACTGTTTTCCTGCCTTGGTG	GACGTGACACAACCAGCATC
MMP28	CAAGCCTGGACGATTTTGTT	ACTGAGCCAGGAGGTTCTGA
ADAM9	GGCACAAGATGTGACAATGG	TCCACACTTCCTCCGTATCC
ADAM10	GGGCAATGTGCAGGTTCTAT	GATGGTTCGACCAACGAAGT
ADAM15	GGCCTCACACTGGACAATTT	GATGCAACTCCCAGGACACT
ADAM17	CTGTGGTGCAAAAGCAGAAA	CTGCCTAATGCCTCCCAATA
ADAM18	GACCATGCTGCACATCAAAC	CAATACGCAGTTCCCAACCT
ADAM22	CCAGGTATGCAGAAGGTGGT	GACACAGCAGGCAAGAATGA
ADAM23	AAGAACCCCTCTCACCACCT	CCACTGGAGTGTGTCATTGG
ADAM28	TGTGTGATGGCAAATCTGGT	ATCCACCTTCATTCCTGCTG
ADAM33	CCCCATGTGGACCTAGAATG	ACCAAATCCTGGCTTGTCAC
RIG-I	AGAGTGGTGGAGGAATGCCA	GTTCCGTCTGGCACAGAAGAT
TLR3	AGCAAGTTGAAGCTGGCTGT	CTGCGATCTCGAAAGTGAAACATC
