# Supplementary material for: Wnt activation disturbs cell competition and causes diffuse invasion of transformed cells through NF-κB-MMP21 pathway
Source: Nat Commun. 2023 Nov 3;14:7048. doi: 10.1038/s41467-023-42774-6 (PMC10624923; doi:10.1038/s41467-023-42774-6)
Supplement: Supplementary file 3 — Description of Additional Supplementary Files [file 41467_2023_42774_MOESM3_ESM.pdf]

Description of Additional Supplementary Files

Supplementary Data 1

A list of differentially expressed genes in β-cat ΔN/RasV12 cells co-cultured with β-cat ΔN cells compared to β-cat ΔN/RasV12 cells cultured alone.
